# Construction of phosphorylation interaction networks by text mining of full-length articles using the eFIP system

**DOI:** 10.1093/database/bav020

**Published:** 2015-03-31

**Authors:** Catalina O. Tudor, Karen E. Ross, Gang Li, K. Vijay-Shanker, Cathy H. Wu, Cecilia N. Arighi

**Affiliations:** ^1^Department of Computer and Information Sciences and ^2^Center for Bioinformatics and Computational Biology, University of Delaware, Newark, DE, USA

## Abstract

Protein phosphorylation is a reversible post-translational modification where a protein kinase adds a phosphate group to a protein, potentially regulating its function, localization and/or activity. Phosphorylation can affect protein–protein interactions (PPIs), abolishing interaction with previous binding partners or enabling new interactions. Extracting phosphorylation information coupled with PPI information from the scientific literature will facilitate the creation of phosphorylation interaction networks of kinases, substrates and interacting partners, toward knowledge discovery of functional outcomes of protein phosphorylation. Increasingly, PPI databases are interested in capturing the phosphorylation state of interacting partners. We have previously developed the eFIP (Extracting Functional Impact of Phosphorylation) text mining system, which identifies phosphorylated proteins and phosphorylation-dependent PPIs. In this work, we present several enhancements for the eFIP system: (i) text mining for full-length articles from the PubMed Central open-access collection; (ii) the integration of the RLIMS-P 2.0 system for the extraction of phosphorylation events with kinase, substrate and site information; (iii) the extension of the PPI module with new trigger words/phrases describing interactions and (iv) the addition of the iSimp tool for sentence simplification to aid in the matching of syntactic patterns. We enhance the website functionality to: (i) support searches based on protein roles (kinases, substrates, interacting partners) or using keywords; (ii) link protein entities to their corresponding UniProt identifiers if mapped and (iii) support visual exploration of phosphorylation interaction networks using Cytoscape. The evaluation of eFIP on full-length articles achieved 92.4% precision, 76.5% recall and 83.7% F-measure on 100 article sections. To demonstrate eFIP for knowledge extraction and discovery, we constructed phosphorylation-dependent interaction networks involving 14-3-3 proteins identified from cancer-related versus diabetes-related articles. Comparison of the phosphorylation interaction network of kinases, phosphoproteins and interactants obtained from eFIP searches, along with enrichment analysis of the protein set, revealed several shared interactions, highlighting common pathways discussed in the context of both diseases.

**Database URL**: http://proteininformationresource.org/efip

## Introduction

Protein phosphorylation is a reversible post-translational modification (PTM) where a phosphate group is added to a protein by a protein kinase. Protein interaction data involving phosphorylated proteins are critical for understanding protein networks and prediction of functional outcomes. Proteins can be phosphorylated on different residues, leading to either activation or down-regulation of their activities, alternative subcellular locations and/or interaction with distinct binding partners. Knowledgebases that curate protein–protein interactions (PPIs) are becoming more interested in capturing context information, such as the phosphorylation state of interactants. In particular, a curation effort is under way in the Protein Ontology (PRO) (1) to create ontology terms for phosphorylated proteoforms and to curate their properties, including PPIs. To facilitate this, we have previously developed a text mining tool, called eFIP—Extracting Functional Impact of Phosphorylation (2). We have noticed that protein interaction data involving phosphorylated proteins are abundant in the scientific literature and can be automatically extracted with high accuracy using pattern-based rules. An example of a positive sentence for eFIP looks like this:

‘These data suggest that **Bax Thr167 is phosphorylated** in response to cytokine treatment, and that **Thr167 phosphorylation** *facilitates* **Bax binding to Pin1**’****. (PMC 2847832)

The phosphorylation and interaction mentions are emphasized in bold, and the impact of phosphorylation on the interaction is emphasized in italics. From this sentence, eFIP extracts ‘Bax’ as the substrate (phosphorylated protein), ‘Thr167’ as the site of phosphorylation, ‘Pin1’ as the interacting protein and ‘facilitates’ as the impact of phosphorylation on the PPI.

eFIP works by first detecting mentions of phosphorylation and PPIs involving the phosphorylated protein. The types of PPIs captured by eFIP include interactions between two proteins, or interactions between a protein and a protein complex, protein region or protein class. Once the phosphorylation and PPI mentions are detected, the second step is to identify a possible relation between the two events.

The work reported in this article was motivated by feedback received during the participation of the eFIP system in the BioCreative-2012 Workshop Track III—Interactive Text Mining (3). eFIP testing was extended to researchers and scientists other than those curating information for the PRO (2).

Addressing the users’ feedback, the main contributions of this work are: (i) full-scale processing of full-length articles from the PubMed Central open-access (PMC OA) database; (ii) the enhancement of the PPI module to include additional words/phrases for PPIs; (iii) the inclusion in the pipeline of iSimp, a sentence simplifier (4, 5) to improve the recall when extracting phosphorylation–PPI relations; (iv) the incorporation of the latest version of the RLIMS-P system [RLIMS-P 2.0 (6, 7)] for phosphorylation event extraction; (v) the enhancement of the website, which allows a user to search for specific kinases, substrates, interacting proteins, keywords or lists of document IDs; (vi) the visualization of the network of interacting proteins via the Cytoscape package (8); (vii) the inclusion of gene normalization via the GenNorm system (9); (viii) an evaluation of the eFIP system on full-length articles and (ix) a corpus of annotated data from 100 randomly chosen sections from full-length documents, containing 272 unique annotations.

We will first describe the eFIP system, then present the results of the system evaluation on the information extracted from PMC OA full-length articles. Finally, we will highlight aspects of the user interface and provide a use case scenario based on phosphoproteins that interact with 14-3-3 protein family members.

## Related work

To the best of our knowledge, there is no other system that extracts the same information as eFIP as a whole. However, various systems address individual tasks.

For the detection of phosphorylation events, we acknowledge MinePhos (10), the work of Veuthey *et al.* (11), the work of Šarić *et al.* (12), and the systems that participated in the BioNLP 2011 Shared Task (13). We chose the RLIMS-P system (6, 7) because it has been evaluated with a corpus covering a wide variety of expressions describing phosphorylation events; it extracts information from multiple sentences; and it has recently been improved with new generalizable rules that boost its performance and allow for the possibility of extending to other post-translational modifications.

For the detection of PPI events, we acknowledge the systems described by Papanikolaou *et al.* (14), as well as the AkaneRE system (15) and SUISEKI (16). Four of these systems are currently available as stand-alone [BioRAT (17), openDMAP (18), PPIExtractor (19) and PPLook (20)], with two of them available when we started creating the eFIP system. However, we could not easily integrate them into our pipeline to address our needs, where specific types of PPIs of interacting proteins were sought. For this reason, we have developed our own in-house system, which is trigger based and uses pattern-based rules.

For the detection of temporal and causal relations between events, we acknowledge the works of Lapata and Lascarides (21), Mani *et al.* (22), Girju (23), Blanco *et al.* (24), Raghavan *et al.* (25), Miwa *et al.* (26), van der Horn *et al.* (27) and Mihăilă *et al.* (28). Because eFIP focuses on a particular type of temporal and causal relation between phosphorylation and PPI events involving the phosphorylated protein, these more general approaches could not be used without considering additional rules specific to this case.

## Materials and methods

**Figure 1.**
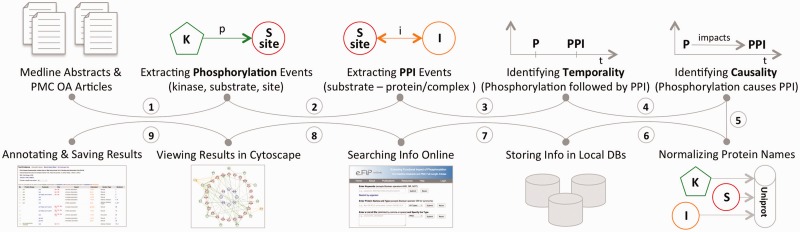
The pipeline of the eFIP text mining system: both abstracts and open-access full-length articles are inspected for phosphorylation (P) mentions (1), PPI events (2) and the temporal/causal relationships between these two events (3, 4). All the proteins involved in the phosphorylation and the PPI events are normalized to the UniProtKB (5), and the information is stored in local databases (6). Users can search and browse the information via a web interface (7), see the results in Cytoscape view (8) or download the information in CSV (Comma Separated Value) file format (9).

This section describes the three distinct aspects of the eFIP method: (i) the eFIP extraction system; (ii) the online interface and (iii) the development of a use case. The first two steps are shown in Figure 1. MEDLINE abstracts and PMC OA articles were used for the full-scale mining of phosphorylation and PPI events. Phosphorylation events were detected first, followed by PPIs that involve the phosphorylated proteins. Because eFIP aims to extract the impact of phosphorylation on PPIs involving the substrate, we next determined the chronological order of the two events and then extracted a potential cause-effect relationship. All proteins involved in the phosphorylation and PPI events were normalized to the UniProt knowledgebase, and the information was stored in a local database. To search, browse, visualize and download the text-mined results, we created a publicly available website: http://proteininformationresource.org/efip.

### Extracting phosphorylation events

For this step, we used the RLIMS-P 2.0 system (6, 7). RLIMS-P is a rule-based phosphorylation information extraction system that identifies tuples of the form <kinase, substrate, site>. For example, the tuple **<Akt, Chk1, Ser280>** is extracted by RLIMS-P from the following sentence:

‘[**Chk1**]_*substrate* is directly [**phosphorylated by**] [**Akt**]_*kinase* at [**Ser280**]_*site*, a modification that results in cytoplasmic sequestration’. (PMC 3317191)

RLIMS-P employs techniques that combine information found in different sentences, because rarely are the three objects (kinase, substrate and site) found in the same sentence. The system itself is available as a stand-alone tool for text mining at the following URL: http://research.bioinformatics.udel.edu/rlimsp.

### Extracting PPI events

For the extraction of PPI events, we are using an in-house rule-based tool, which extracts interactions between proteins, as well as interactions between a protein and a protein complex, protein region or class of proteins. The PPI tool extracts tuples of the form <first_interactant, second_interactant, type_of_PPI>. For example, **<BIM_EL_, BAX, interaction>** is extracted by the PPI tool from the following sentence:

‘BSO induces the [**interaction**]_*type_of_PPI* of phosphorylated [**BIM_EL_**]_*interactant* with [**BAX**]_*interactant*’*.* (PMC 4029189)

The tool is trigger based, meaning that only the sentences containing mentions of specific types of interactions are considered for the extraction of binding proteins. Although previously, the extraction of PPIs was based on the following trigger words: ‘binding’, ‘interaction’, ‘complex’, ‘association’, ‘dissociation’, ‘dimerization’ and their textual variations, in this work, we have enhanced the PPI tool to extract PPIs described with additional trigger words: ‘affinity’, ‘precipitation’, ‘recruitment’, ‘release’ and ‘sequestering’, as well as their textual variations. For more information about this tool, we refer the reader to our previous work (2).

### Extracting phosphorylation → PPI impact

The impact module was described in detail in a previous publication of the eFIP system (2). Here, we will briefly summarize with examples, and concentrate on the addition of iSimp (4, 5) for sentence simplification.

The goal of the impact module is to find information about the ability of phosphorylated proteins to interact with other proteins. For this, we first need to find, in the same sentence, mentions of a phosphorylation event and mentions of a PPI event involving the phosphorylated protein. The next step is to detect whether the phosphorylation event occurs before the PPI event (temporal relation) and, if so, whether the phosphorylation event is causing any change in the PPI event (causal relation). A simple temporal relation can be seen in the following sentence:

‘Phosphorylated **BIM_EL_** was dissociated from **MCL1** and interacted with **BAX**’****. (PMC 4029189)

A more complex sentence about phosphorylated BIM_EL_ shows how the interaction with MCL1 is disrupted after their phosphorylation, while the interaction with BAX is increased after the phosphorylation:

‘Addition of BSO induced mitochondrial injury-mediated apoptosis via the phosphorylation of **BIM_EL_** and **MCL-1**, ***resulting in*** their dissociation, and ***increased*** the interaction between **BIM_EL_** and **BAX**’****. (PMC 4029189)

The above sentence is complex for two reasons: (i) the dissociated proteins are referred by the pronoun ‘their’, which calls for a reference resolution between ‘their’ and ‘BIM_EL_’ and ‘MCL-1’; and (ii) one phosphorylation event affects two PPI events mentioned in a conjunction (i.e, the dissociation and the interaction, connected via the conjunction ‘and’). We could design a syntactical pattern that looks for a phosphorylation event impacting two PPI events in a sentence. However, this would not be the best solution, because each unique complex sentence would require the design of a unique syntactical pattern to extract the information from the sentence, and given the variety in writing styles, the number of such patterns could grow exponentially.

For this reason, we decided to concentrate only on a few simple syntactical patterns that yield high precision on simple syntactical constructions, and break complex sentences into multiple simple sentences to improve the recall. When run on the above sentence, our tool, iSimp, generates the following three simple sentences.

1. Addition of BSO induced mitochondrial injury-mediated apoptosis via the phosphorylation of **BIM_EL_** and **MCL-1**.
2. The phosphorylation of **BIM_EL_** and **MCL-1 *results in*** their dissociation.
3. The phosphorylation of **BIM_EL_** and **MCL-1 *increased*** the interaction between **BIM_EL_** and **BAX**.

Using simple patterns, the impact module will extract from the second sentence that the phosphorylation results in dissociation, and from the third sentence that the phosphorylation increases the interaction. iSimp detects not only conjunctions, but also lists, relative clauses, appositives, as well as some situations in which these constructs are nested. This is explained in detail in the works of Peng et al. (4, 5).

### Linking protein names to UniProtKB

Normalization of protein names (kinases, substrates and interactants) was performed using the GenNorm system (9). This step is new in this version of eFIP. GenNorm was evaluated as part of the BioCreative III, ranking second. The results were reported as 46.56 F-measure on a gold standard set, and 55.09 F-measure on a silver standard set of 507 articles. We have also performed a local evaluation of GenNorm. On a set of 100 randomly selected abstracts, we obtained very similar results to the ones reported as part of BioCreative III. However, when evaluated on a set of 100 abstracts randomly selected from the ones mentioning phosphorylation information, GenNorm performed much better, with precision of 93.5, recall of 66 and F-measure of 77.4.

### Large-scale processing

**Figure 2.**
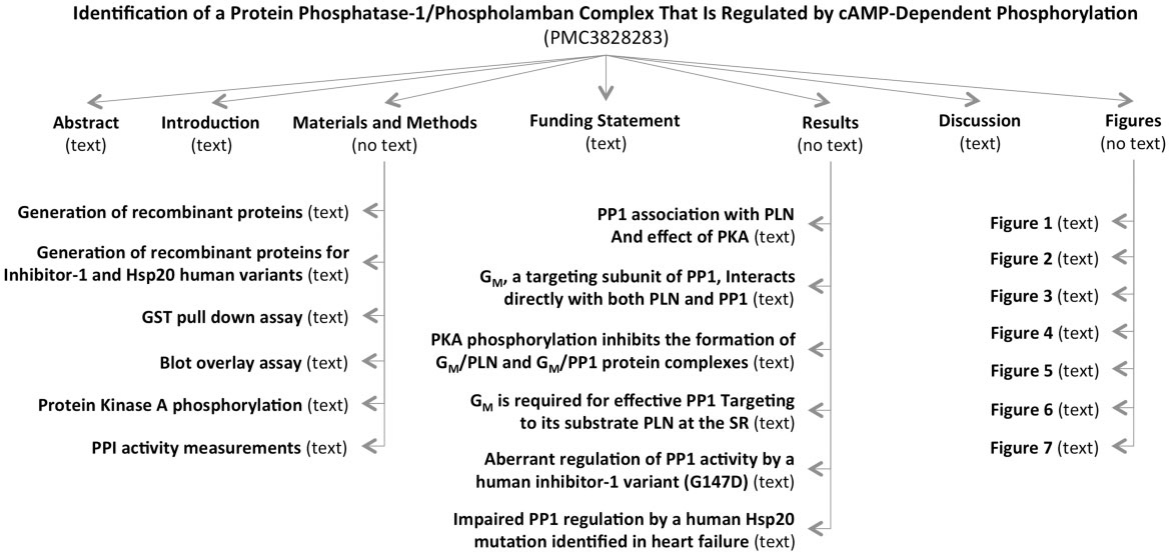
Example of sections and subsections in an article. There are as many nodes as headings in an article. However, the number of actual subsections in an article can be lower if no text is found between two headings (e.g., Results subsection does not exist because there is no text under the Results heading and before the ‘PP1 association with PLN and effect of PKA’ heading).

MEDLINE abstracts and PMC OA articles were indexed locally for quick retrieval using an in-house adaptation of the Lucene indexer (29). The numbers presented throughout this article reflect the corpora as of September 2014. A total of 21 900 340 non-empty abstracts and 576 494 non-empty full-length articles were successfully indexed. The PMC articles were further split into subsections, as shown in Figure 2, yielding a total of 12 357 367 subsections (or 21.5 subsections per article), which varied in type (e.g., introduction vs. results vs. figure caption).

An in-house XML parser was written to split the articles into subsections, as our module for extracting phosphorylation events was written for abstracts (or, in general, short sections containing all the necessary information for the phosphorylation event). Thus, we wanted to generalize our code from abstracts to other types of sections in an article. We noticed other advantages for splitting an article into subsections, mostly having to do with the confidence and certainty associated with the information extracted from various types of subsections. For example, information extracted from a sentence coming from a Results subsection might be marked with a much higher confidence score than information extracted from a sentence coming from the Introduction section. These confidence scores can then be used to display the information extracted from one article in a ranked list, or to solve potential conflicts across papers. We will pursue assigning confidence scores in the near future.

**Figure 3.**
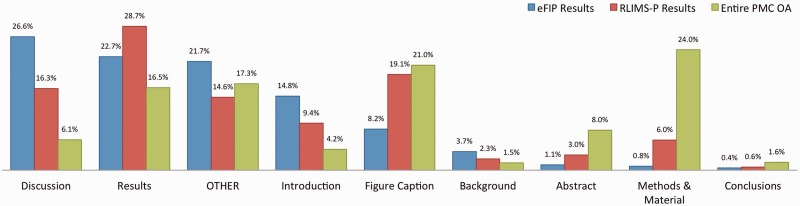
Distribution of subsections in the eFIP results (blue), RLIMS-P results (red) and the entire PMC OA collection (green).

In our work, a subsection is considered to be any heading together with the text occurring right afterwards and before the next heading in the article. For example, the ‘Abstract’ subsection is composed of the text between the Abstract heading and the ‘Introduction’ heading. Likewise, the heading ‘GST pull down assay’ and the text between the ‘GST pull down assay’ and ‘Blot overlay assay’ headings in Figure 2 constitute a subsection. To each subsection, we also associate the titles/headings of the parent nodes, as these could hold important information complementing the actual text. Thus, the title of the article itself is always listed together with every subsection, and the title of major sections, such as ‘Results’ is also carried over in the subsections. A type is assigned to each subsection, depending on the title of the subsection’s parent, or based on the type of subsection specified in the original XML file. If no type can be determined, either because no annotation could be found in the XML file, or because the parent section has a title other than the typical Introduction, Results, Discussion and so on, then a type of ‘other’ is assigned to the subsection. The types of subsections and their distribution in the local PMC index are as follows: Abstract (8%), Introduction (4.2%), Background (1.5%), Methods & Materials (24%), Results (16.5%), Discussion (6%), Conclusions (1.6%), figure (21%) and other (17.2%). This can be seen in Figure 3.

### Creation of corpus for evaluation

For the evaluation of full-length documents, we selected 100 subsections from PMC OA articles. Subsections were chosen, one by one, in a random fashion. Some of the subsections were filtered out, such that the remaining 100 subsections spanned articles from 2000 to 2014 and followed to some extent the distribution of eFIP results by subsection. This selection process resulted in 24 Results, 21 Discussion, 18 Other, 17 figure captions, 13 Introduction, 5 Background and 2 Methods and Materials sections. The abstract sections were not considered in this evaluation, because they were evaluated in the previous work.

The subsections were shown to three biocurators of the PRO database, who are familiar with the output format of the eFIP system. All three biocurators have doctoral degrees in biosciences. The biocurators were not given access to the results of the system nor were they allowed to look at the eFIP results, in order to avoid any potential bias. An initial test set given to all three biocurators revealed that inter-annotator agreement was 100%. Thus, the annotators were given different subsections to annotate, but encouraged to discuss the results among themselves when in doubt. Two biocurators annotated 25 subsections each, and the other annotated 50 subsections. On average, each biocurator spent 2 h for every 10 subsections. A total of 20 h was spent cumulatively for the entire corpus. The gold standard corpus contains information about the phosphorylation events (kinase, substrate site) and the interaction events (interacting partner of the substrate, type of PPI, type of relation) found in each subsection, and it can be downloaded from http://research.bioinformatics.udel.edu/eFIPonline/Corpus.zip.

### 14-3-3 Proteins use case

eFIP was queried via the eFIP web interface using the search/query terms ‘14-3-3 AND cancer’ and ‘14-3-3 AND diabetes’. Results were viewed by interactant, and those results where the interactant was 14-3-3 or one of the 14-3-3 isoforms (e.g., 14-3-3beta) were validated by manual inspection of the accompanying text evidence. Redundant results were merged. The list of unique interactions is provided in supplementary Table S1. Network views were created using Cytoscape 3.1.1 (30). KinBase was used to identify kinase families (31). Functional annotation clustering was performed using the DAVID web interface (http://david.abcc.ncifcrf.gov/) (32) with ‘high’ classification stringency and all other parameters with default settings. Clusters with enrichment scores >2.5 and terms within those clusters with Benjamini–Hochberg score <0.05 were selected for display in the treemap. Names for the clusters were chosen based on manual assessment of the predominant process/pathway among the terms in the cluster. The treemap was created using the R (version 3.0.3; http://www.r-project.org/) treemap function. Information about the enriched terms, including *P* values and associated genes, is provided in supplementary Table S2.

### Evaluation Metrics

The accuracy of eFIP, with respect to document-level and information extraction, was evaluated in terms of precision (*P*), recall (*R*) and F-measure (*F*). We define these measures here:

$$P=\frac{\text{TP}}{\text{TP}+\text{FP}},R=\frac{\text{TP}}{\text{TP}+\text{FN}},F=\frac{\text{2}*\text{Precision}*\text{Recall}}{\text{Precision}+\text{Recall}}$$

where true positive (TP) is the number of documents/information correctly found to be positive by eFIP, true negative (TN) is the number of documents/information correctly found by eFIP to be negative, false positive (FP) is the number of documents/information that eFIP mistakenly tags as positive and false negative (FN) is the number of documents/information that eFIP fails to tag as positive.

## Results and discussion

In this section, we present the following: (i) the statistics on full-length articles processed in eFIP; (ii) the results of the evaluation of eFIP on full-length articles; (iii) a description of the enhanced eFIP web interface and (iv) a use case exploring 14-3-3 proteins and their phosphorylated binding partners in two disease contexts.

### Statistics on eFIP results in full-length articles

Approximately 67 084 PMC articles and 220 628 MEDLINE abstracts contain phosphorylation triggers. Of those, 77.6% of all PMC articles and 74.7% of all MEDLINE abstracts were found to contain phosphorylation events using the RLIMS-P system. About 5866 PMC articles (or 19 341 subsections) and 7441 MEDLINE abstracts contained information about PPIs involving phosphorylated proteins, which yielded a total of 23 244 sentences with phosphorylation–PPI information. Using the GenNorm system, we were able to link proteins to 6861 unique UniProtKB entries in PMC articles (2610 unique kinases, 6281 unique substrates and 2469 unique interacting proteins), as well as 16 195 unique UniProtKB entries in MEDLINE abstracts (5516 unique kinases, 15 086 unique substrates and 6040 unique interacting proteins).

Figure 3 shows the distribution of results extracted by eFIP from full-text articles for the various types of subsections. A total of 7877 subsections (spanning 5866 different articles) contain phosphorylation–PPI relations that are relevant to eFIP: 2098 (26%) Discussion subsections, 1784 (23%) Results subsections, 1166 (15%) Introduction subsections, 646 (8%) figure captions, 292 (4%) Background subsections, 85 (1%) Abstracts, 64 (1%) Methods and Materials subsections, 34 (<1%) Conclusion subsections and 1708 (22%) other subsections. Of the 5866 eFIP-positive full-length articles, 5789 (98.7%) did not contain any information in the abstract sections, suggesting that there are many more articles in MEDLINE that would be eFIP positive if the full-length article were available. A similar result was observed for RLIMS-P-positive articles, with 47 822 (91.9%) of the 52 063 total articles lacking phosphorylation information in the abstract.

### Evaluation of PMC subsections

A total of 272 annotations were marked as eFIP positive by the biocurators, i.e., containing a phosphorylation–PPI relation of interest to eFIP, with 77 containing temporal relations and 195 containing causal relations. Substrate and interacting protein information was annotated in all 272 tuples. However, only 118 of the annotations contained kinase information, and only 134 contained phosphorylation site information. We have conducted two evaluations, as shown in Table 1.

**Table 1.** Results of the eFIP evaluation

Evaluation type	All	TP	TN	FP	FN	P	R	F
Subsection level	100	89	4	7	0	**92.7**	**100.0**	**96.2**
Information level	289	208	Not applicable	17	64	**92.4**	**76.5**	**83.7**

First, we looked at the number of subsections correctly identified by the eFIP system to contain relevant <kinase, substrate, site, interactant, relation type> tuples. For a curator, presenting relevant articles or subsections might be sufficient to aid in the curation process. Ninety-three of 100 subsections were correctly identified as positive or negative with respect to the gold standard, and 7 subsections were incorrectly identified as positive. This resulted in a precision of 92.7, recall of 100 and F-measure of 96.2 at the subsection level.

Second, we looked at the amount of information correctly identified in these subsections. For this, we considered all the unique tuples identified by eFIP and/or marked by the annotators. From a total of 272 unique <kinase, substrate, site, interactant, relation type> tuples marked by the curators, eFIP correctly identified 208 as positive (TP), and missed 64 tuples (FN). Additionally, it incorrectly identified 17 as positive (FP), thus giving us a total of 289 unique tuples for evaluation. This resulted in a precision of 92.4, recall of 76.5 and F-measure of 83.7.

Note that we have evaluated eFIP as an end-to-end system. Any error in part-of-speech tagging, parsing or by one of the components (e.g., RLIMS-P, the PPI module, iSimp or the impact module) will likely cause an eFIP error. All the false positives (17) are attributed to the impact module, where the directionality was, in fact, the opposite (i.e., interaction event first and phosphorylation event second) or non-existent. Writing styles in the full-text articles are different from those in abstracts (33). We observed many complex sentences that were beyond the grasp of our sentence simplifier, nesting a large number of constructs and combining various phosphorylation and PPI events. We attribute 28 recall errors to highly complex sentences. The remaining false negatives were due to RLIMS-P not extracting the phosphorylation event or substrate correctly (15), the PPI module failing to identify the PPI event or the correct interacting protein (16) and the impact module failing to detect an impact (5). In ∼85% of tuples that eFIP missed, the information was mentioned only once in the entire subsection, suggesting that whenever information is mentioned multiple times throughout a subsection, eFIP is almost always able to extract it. This is not to say that eFIP correctly extracts information only when it is mentioned multiple times in a subsection. In fact, 134 of 208 true positive tuples were mentioned only once in their respective subsections.

### Web interface and user interaction

**Figure 4.**
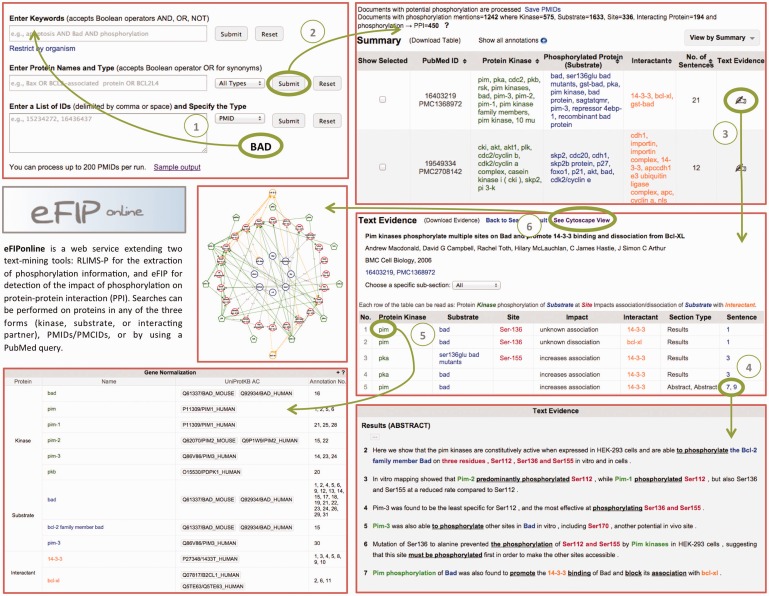
Snapshot of eFIP web interface. The Search Page (top left) allows for various search criteria. The Results Page (top right) displays statistics about amount of documents, kinases, substrates, sites and interacting proteins found for the search criteria, together with a table of the information extracted, which can be displayed in various views. The Document Page (middle right) shows information about the document: title, authors, journal, IDs, as well as a table of the information extracted from the document. The Document Page continues with the gene normalization information (bottom left) and the actual sentences highlighted with corresponding colors for kinase, substrate, site, interactant and trigger words (bottom right). A Cytoscape view (middle left) of the network of interacting proteins from the documents can be seen in a separate window.

After the participation of eFIP in the BioCreative-2012 Workshop Track III—Interactive Text Mining (3), we have concentrated our efforts on user satisfaction regarding the interaction with the web interface. The new website consists of five main pages: the Homepage (or Search Page), the Summary Page (or Results Page), the Text Evidence Page (or Document Page), the Cytoscape View Page and the Login Screen facilitating validation of results. Screenshots of the website are shown in Figure 4.

The Search Page allows for various search criteria. Keywords or phrases can be combined using Boolean operators. This input is used to query PubMed, and resulting documents are then intersected with the results stored in our local database. Users can search for a protein together with its role (kinase, substrate, interacting partner or any role). Because a protein is known by many names, we allow for the search of all synonyms, delimited by the Boolean operator OR. An example query is ‘Bax OR Bcl2-associated protein OR BCL2L4’. Note that users can search for multiple proteins this way. Alternatively, users can also provide a list of PMIDs (PubMed Identifiers) or PMCIDs (PubMed Central Identifiers), delimited by comma, space or new line.

At the top of the Results Page, one can see statistics on the numbers of documents, kinases, substrates, sites and interacting proteins found for the search criteria. The document IDs (Identifiers) can be downloaded if needed. The actual results are displayed in a tabular format below the statistics. Different views of the results are available: by kinase, by substrate, by interactant or by the document ID. The results displayed in the table can be downloaded, and the order of the information in the comma-delimited file will vary depending on the view from which it was downloaded.

Clicking on the ‘Text Evidence’ icon will bring the user to the Document Page. Basic information about the document is provided at the top of this page: the title, the authors, the journal, the year of publication and the document IDs. If the document is a full-length article, then the various sections are listed in a drop-down list, so that the user can choose to concentrate only on one subsection, as opposed to the entire document. The kinases, substrates, sites, interactants, subsection types, impact details and sentence numbers are shown in tabular format. Clicking on any of the rows will highlight the actual sentence, below, from which the information was extracted. The sentences are displayed at the bottom of the page, with the relevant information colored the same way as in the table (green for kinases, blue for substrates, red for sites and orange for other interacting proteins). Whenever possible, proteins are also normalized to UniProt identifiers and displayed below the table of results.

Clicking the ‘See Cytoscape View’ link at the top of the page will open a new window, with all the proteins (kinases, substrates, interacting partners) displayed in a graph. Kinases are shown with green pentagons, and the arrows representing phosphorylation events are drawn in green. Substrates and corresponding sites are shown in red circles. These are connected by gray lines to the corresponding un-phosphorylated proteins, shown in blue circles. Finally, the interacting proteins are shown in orange circles, connected to the phosphorylated protein by straight orange lines in case of an association and dashed orange lines in case of a dissociation. Whenever the impact suggests an increase in the association of the two proteins, a ‘+’ is displayed. Conversely, a ‘−‘ is displayed when the impact suggests a decrease in the association of the two proteins. Hovering over any node in the graph will highlight only the nodes and edges connected with the initial node by a distance of maximum two. The Cytoscape graph can be downloaded as a PNG image or in XGMML format, which is Cytoscape compatible.

To validate the results, provide additional annotations and/or leave comments, users need to log in. Once logged in, this feedback can also be saved and retrieved at a later time for further edits. Gene normalizations can also be validated. If an identifier is incorrect, the annotator can link the protein to the correct UniProt ID.

### Interactions of 14-3-3 proteins in a disease context

To demonstrate the use of the eFIP system, we explored phosphorylation-dependent interactions involving 14-3-3 proteins. 14-3-3 proteins modulate a range of cellular processes, including cell proliferation and programed cell death, through their interactions with Ser/Thr-phosphorylated domains of other proteins (34). The effect of 14-3-3 proteins on their binding partners varies. In some cases, 14-3-3 proteins inhibit the catalytic activity of the interacting protein or its access to substrates [e.g., CDC25 (35)]; in other cases, they potentiate protein activity [e.g., WEE1 (36)]; and in still other cases, their effects are more complex. For example, 14-3-3 binding to the cyclin-dependent kinase (CDK)-inhibitor CDKN1B (p27 Kip1) positively regulates the protein by protecting it from ubiquitin-mediated degradation, but also negatively regulates it by sequestering it in the cytoplasm away from its nuclear targets (37, 38). In keeping with their key role in regulating the cell cycle, 14-3-3 proteins have been implicated in cancer (34); aberrant 14-3-3 interactions have also been linked to Alzheimer’s disease (39) and diabetes (40).

**Figure 5.**
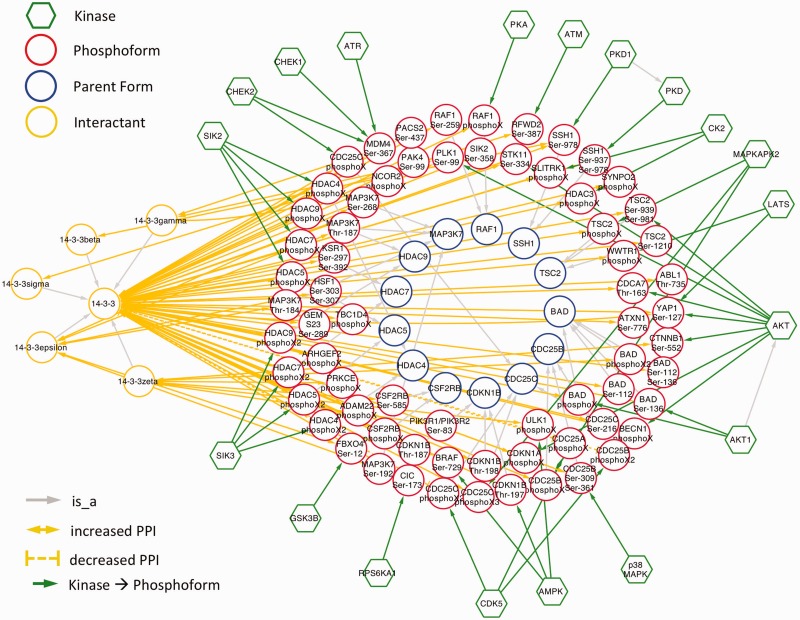
Network showing eFIP results from cancer-related papers where 14-3-3 is the interactant.

Because 14-3-3 interactions are regulated by phosphorylation, we would expect them to be well-represented among eFIP results. Indeed, querying eFIP with ‘14-3-3’ returns 814 phosphorylation-dependent PPIs in 1242 articles with phosphorylation mentions. Taking advantage of the keyword search feature of eFIP, we next investigated 14-3-3 interactions mentioned in the context of two diseases, cancer and diabetes. Searching for 14-3-3 AND cancer returns 187 phosphorylation-dependent PPIs in 280 articles with phosphorylation mentions. After restricting to cases where 14-3-3 or one of its isoforms is the interactant and merging redundant results, there were 70 interactions involving 69 phosphoforms of 45 unique proteins and 14-3-3 or one of its isoforms (beta, gamma, epsilon, zeta or sigma) (Figure 5 and supplementary Table S1). Of the 45 proteins, 15 (33%) were not included in an in-depth manual literature curation of 14-3-3 binding proteins (41). With one exception, the 14-3-3 binding partners that were not included were discovered after the publication date of the study (2010), thus underscoring the need for automated text-mining tools such as eFIP to keep up with the ever expanding volume of scientific knowledge. Importantly, because the eFIP interactions were extracted from articles that were retrieved by a search for the keyword ‘cancer’, they are likely to represent the subset of 14-3-3 interactions that are relevant in the context of this disease.

**Figure 6.**
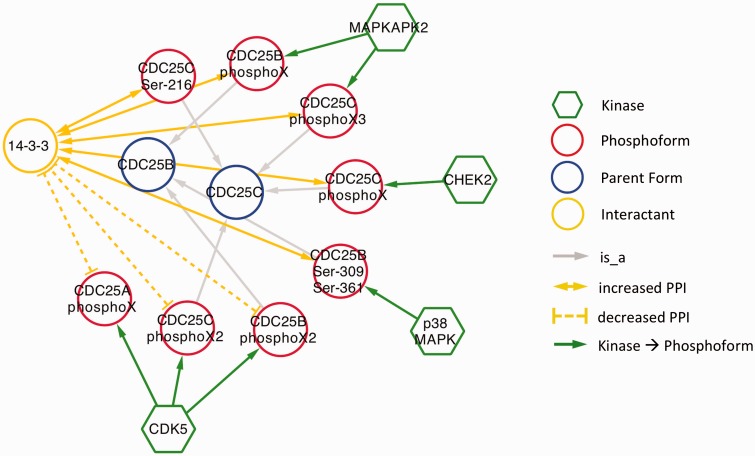
Interactions of CDC25 phosphoforms with 14-3-3 proteins in a cancer context.

As expected, in nearly all cases, phosphorylation promoted association with 14-3-3. The exception was CDK5 phosphorylation of CDC25A, CDC25B and CDC25C (Figure 6). CDC25 proteins, which are phosphatases that drive cell cycle progression by removing inhibitory phosphorylations from CDKs exhibit multiple, complex interactions with 14-3-3 proteins (34). The interactions identified in our eFIP cancer-focused search are shown in Figure 6. In addition to CDK5-dependent phosphorylation of CDC25B, which reduces binding to 14-3-3, CDC25B is phosphorylated by two MAP kinase pathway members (MAPKAPK2 and p38 MAPK), resulting in enhanced association with 14-3-3. Similarly, CDC25C phosphorylated by p38 MAPK or the DNA damage-induced kinase CHEK2 shows enhanced binding to 14-3-3. It should be noted that each mention of phosphoprotein with no site information was treated as a separate phosphoform. For example, the MAPKAPK2-phosphorylated form of CDC25B, whose phosphorylation sites were not mentioned, is treated as a separate phosphoform (CDC25B phosphoX) from the p38 MAPK-phosphorylated form (CDC25B Ser-309 Ser-361). Given that both phosphoforms show enhanced binding to 14-3-3 and that 14-3-3 proteins recognize a conserved phosphorylated motif, it is quite possible that the two forms are in fact phosphorylated on the same sites.

For ∼60% of the interactions, kinase information was also extracted. In addition to cases where the kinase directly phosphorylated the 14-3-3 binding protein, we included cases where there was ambiguity in the text as to whether the kinase was directly or indirectly involved (supplementary Table S1). For example, based on this sentence: ‘Third, ATM-mediated phosphorylation of COP1 at S387 promotes COP1's binding to 14-3-3σ’ (42), ATM kinase activity could be directly or indirectly involved in phosphorylation of COP1 (RFWD2). Altogether, 18 kinases that promote 14-3-3 binding and one kinase (CDK5) that inhibits 14-3-3 binding were identified. A recent study found that kinases involved in promoting 14-3-3 binding belonged to two kinase families: AGC and CAMK (41). Of the 18 kinases we found, 5 belong to the AGC family and 8 belong to the CAMK family. In addition, we found two kinases (ATM, ATR) from the atypical family and three kinases (CK2, GSK3B and p38 MAPK) from the CMCG family. Two of the CMCG kinases (CK2 and GSK3B) appear to be directly involved in regulating 14-3-3 binding by phosphorylating the histone deactylase HDAC3 and the F-box protein FBXO4, respectively. Thus, the CMCG kinase family may also play an important role in 14-3-3 interactions.

By far, the most frequently occurring kinase was AKT, which was responsible for 10 phosphorylation events on 9 unique substrates. Faulty regulation of the PI3K/AKT/mTOR signaling pathway has been strongly linked to multiple kinds of cancer (43). There were also several examples where a phosphoform was associated with several different kinases, demonstrating that phosphorylation-dependent 14-3-3 binding can be a point of convergence for multiple signaling pathways triggered by different stimuli. For example, MDM4 phosphorylation on Ser-367 can be mediated, directly or indirectly, by three DNA damage-induced kinases (ATR, CHEK1 and CHEK2), resulting in its association with 14-3-3. The three kinases are activated by different kinds of DNA damage, with ATR and CHEK1 responding primarily to single-stranded breaks, replication fork collapse and ultraviolet damage, and CHEK2 responding primarily to double-stranded breaks (34).

**Figure 7.**
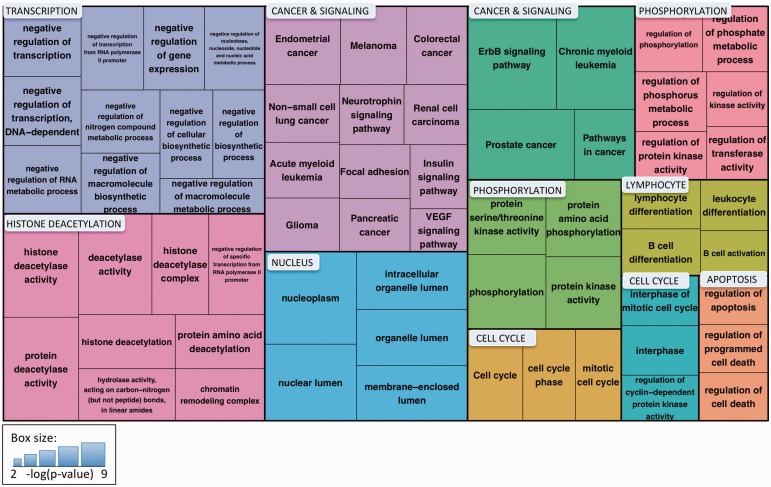
Treemap of enriched Gene Ontology and KEGG pathway terms for proteins identified by eFIP that, when phosphorylated, interact with 14-3-3 proteins in the context of cancer. Enriched terms were clustered using the DAVID Functional Clustering tool, which groups terms based on sharing common genes. Term clusters are represented as different colored blocks. For each term, box size reflects the *p* value of the term enrichment (more significantly enriched terms are in larger boxes). The *p* values range from 6.44 × 10^−9^ (hsa04012:ErbB signaling pathway) to 6.28 × 10^−3^ (hsa04370:VEGF signaling pathway).

To understand the major functional roles of the cancer-associated 14-3-3 binding proteins, we performed Gene Ontology and KEGG-pathway enrichment analysis and clustered the significantly enriched terms using the DAVID Functional Clustering tool, which groups terms based on the assumption that annotations attached to similar sets of genes are likely to be related to each other (32). The results are shown in Figure 7. Of the 11 clusters, 2 were related to cancer and signaling and included terms for 9 different cancer types and several signaling pathways that have been linked to cancer [e.g., ErbB (44) and VEGF (45)]. Other clusters included cell cycle and apoptosis, processes that have been associated with both 14-3-3 function and cancer. Three clusters—transcription (primarily negative regulation of transcription), nucleus and histone deacetylation—point to the important role of 14-3-3 binding partners in regulating gene expression. Two clusters related to phosphorylation reflect the high representation of kinases, phosphatases and kinase regulatory proteins among the 14-3-3 binding proteins. The final cluster includes terms related to B-cell differentiation and activation. Thus, by combining eFIP text mining with term enrichment analysis, we quickly and easily obtained a clear picture of the major processes affected by phosphorylation-dependent 14-3-3 binding in the context of cancer.

**Figure 8.**
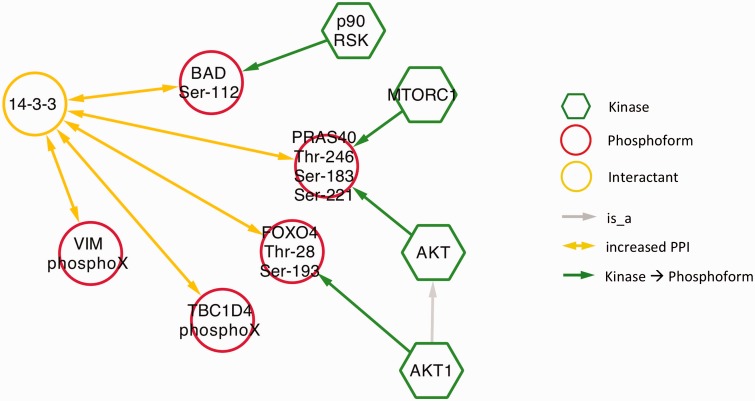
Network showing eFIP results from diabetes-related papers where 14-3-3 is the interactant.

Next, we examined phospho-dependent interactions with 14-3-3 proteins in the context of diabetes by querying eFIP for 14-3-3 AND diabetes. Mentions of 14-3-3 interactions in a diabetes context were much less frequent than in the cancer context. The search returned only 10 phosphorylation-dependent PPIs in 25 articles with phosphorylation mentions. Among these, there were five distinct interactions where 14-3-3 was the interactant (Figure 8 and **s**upplementary Table S1). Four kinases were identified as phosphorylating three of the five phosphoforms. Interestingly, despite the overall small number of interactions, there was considerable overlap with the cancer results. Two of the diabetes-related phosphoforms—BAD Ser-112 and TBC1D4 (AS160) PhosphoX—also appeared in the cancer network (Figure 5) and AKT kinase again played a prominent role, phosphorylating two of the phosphoproteins. These commonalities highlight some biological processes shared by the two diseases. Phosphorylation and 14-3-3 binding of BAD suppresses apoptosis, which is relevant to diabetes in the context of survival of pancreatic islet cells as well as to tumor cell survival (46, 47). Interactions of TBC1D4 with 14-3-3 regulate the localization of the insulin-dependent glucose transporter GLUT4; abnormalities in glucose uptake are characteristic of both diabetes and cancer (48, 49). Finally, the AKT pathway regulates cell proliferation in response to nutrient availability and thus is intimately connected to both glucose sensing and growth signals (50).

As exemplified by this use case, eFIP enables the investigation of phosphorylation-dependent PPIs in a particular biological context (e.g., disease) and comparison of interactions across different contexts. Sets of kinases, phosphoproteins and/or interactants obtained from eFIP searches can be further analysed (e.g., using functional enrichment tools) to gain insight into the biological processes affected by phosphorylation-dependent changes in protein interactions. The information collected about the phospho-interactants and 14-3-3 proteins is being added into the curation pipeline of the PRO (1).

## Conclusion and future work

In this article, we described an enhanced version of the eFIP system for the extraction of PPIs of phosphorylated proteins. We have addressed the usability requests from the BioCreative 2012 Interactive Task and evaluated the system on an expertly annotated corpus, which includes representative subsections of full-length articles. With the 14-3-3 use scenario, we demonstrated that with eFIP it is possible to extract detailed information about phosphorylation-dependent PPIs from what would otherwise be a prohibitively large volume of literature to search manually (e.g., >1000 papers returned in a PubMed search for 14-3-3 AND cancer). Also, the ability to filter eFIP results based on keywords facilitates comparisons of phospho-dependent PPIs in different contexts, which can reveal hidden biological connections (e.g., the relations in common between the cancer and diabetes networks).

Various enhancements were performed on a previous version of the eFIP system to improve the user experience and expand the number of results: the inclusion of full-length articles from the PMC OA database; the enhancement of the PPI module to include additional types of PPIs; the inclusion in the pipeline of a sentence simplifier to improve the recall when extracting phosphorylation–PPI relations; the incorporation of an updated version of the RLIMS-P system for phosphorylation event extraction; the creation of a new website to enhance user experience, allowing them to search for specific kinases, substrates, interacting proteins, keywords or lists of document IDs; the incorporation of a graphical view for the network of interacting proteins; the inclusion of gene normalization; an evaluation of the eFIP system on full-length articles and a corpus of annotated data from 100 randomly chosen sections from the full-length documents.

We envision expanding on this work in the future in various ways. First, because the RLIMS-P patterns and rules were recently generalized to other post-translational modifications, including acetylation, ubiquitination and glycosylation, we are planning in the near future to detect the impact of these PTMs on the interactions of affected proteins. We will adapt the rules, if necessary, and process the entire MEDLINE and PMC OA corpora to extract these other types of PTMs. We are also interested in various other consequences of PTMs. One such relation is between PTMs themselves, as can be seen in the following sentence:

‘An instance of PTM cis-crosstalk is the **phosphorylation of Ser10 residue of histone H3**, which subsequently *leads to* **acetylation of Lys14 residue**’****. (PMC 4120686)

Another type of impact that we are currently investigating is the subcellular localization of the affected protein, as can be seen in the following sentence:

**‘Protein kinase IKKbeta-catalyzed phosphorylation of IRF5 at Ser462** *induces* its dimerization and **nuclear translocation in myeloid cells**’****. (PMID 25326418)

With the inclusion of full-length articles, we have noticed cases in which two events are mentioned in different sentences; however, a cause-effect relationship between them could be deduced. Around 15% of all possible phosphorylation–PPI relations in full-length articles fall into this category, although, according to our previous work, the frequency is much lower in abstracts. We will investigate the use of multiple sentences to detect the types of impact proposed above.

Around 12% of phosphorylation–PPI relations were marked as hypothetical or potential by the annotators, meaning that the information was not conveyed by the authors with 100% confidence. We plan to study this type of relation and identify ways to assign confidence scores to the information extracted by the eFIP system to avoid intra-paper and inter-paper conflicts. For example, an intra-paper conflict might be between the information extracted from the following two sentences within the same article: (i) ‘We have investigated whether protein A maintains its interaction with protein B, when phosphorylated at site X’ and (ii) ‘When phosphorylated at site X, protein A dissociated from protein B’. The first sentence is stating an experimental setup scenario for which the results are unknown, and, thus, the sentence has some doubt, while the second sentence is stating an experimental finding, with 100% confidence. Confidence scores for these two types of sentences would suggest to eFIP that the information coming from the second sentence should replace the information extracted from the first sentence.

Finally, we would like to investigate the creation of Cytoscape networks with information coming from multiple articles, integrating confidence information and highlighting the nodes and edges that are most discussed in the literature.

## Supplementary Data

Supplementary data are available at *Database* Online.

Supplementary Data
