# Supplementary material for: Construction of phosphorylation interaction networks by text mining of full-length articles using the eFIP system
Source: Database (Oxford). 2015 Mar 31;2015:bav020. doi: 10.1093/database/bav020 (PMC4381107; doi:10.1093/database/bav020)
Supplement: Supplementary Data [file supp_2015_bav020_index.html]

Construction of phosphorylation interaction networks by text mining of full-length articles using the eFIP system — Supplementary Data 

# Construction of phosphorylation interaction networks by text mining of full-length articles using the eFIP system

## Supplementary Data

files

**Files in this Data Supplement:**

- Supplementary Data - xlsx file
- Supplementary Data - txt file
